# Endothelial-to-mesenchymal transition in tumour progression and its potential roles in tumour therapy

**DOI:** 10.1080/07853890.2023.2180155

**Published:** 2023-03-13

**Authors:** Zeli Yin, Liming Wang

**Affiliations:** a Engineering Research Center for New Materials and Precision Treatment Technology of Malignant Tumors Therapy, Dalian Medical University, Dalian, Liaoning, China; b Engineering Technology Research Center for Translational Medicine, Dalian Medical University, Dalian, Liaoning, China; c Division of Hepatobiliary and Pancreatic Surgery, Department of General Surgery, The Second Affiliated Hospital of Dalian Medical University, Dalian, Liaoning, China

**Keywords:** Tumour microenvironment (TME), tumour-associated endothelial cells (TECs), endothelial-to-mesenchymal transition (EndoMT), tumour progression, tumour-targeted therapy

## Abstract

Tumour-associated endothelial cells (TECs) are a critical stromal cell type in the tumour microenvironment and play central roles in tumour angiogenesis. Notably, TECs have phenotypic plasticity, as they have the potential to transdifferentiate into cells with a mesenchymal phenotype through a process termed endothelial-to-mesenchymal transition (EndoMT). Many studies have reported that EndoMT influences multiple malignant biological properties of tumours, such as abnormal angiogenesis and tumour metabolism, growth, metastasis and therapeutic resistance. Thus, the value of targeting EndoMT in tumour treatment has received increased attention. In this review, we comprehensively explore the phenomenon of EndoMT in the tumour microenvironment and identify influencing factors and molecular mechanisms responsible for EndoMT induction. Furthermore, the pathological functions of EndoMT in tumour progression and potential therapeutic strategies for targeting EndoMT in tumour treatment are also discussed to highlight the pivotal roles of EndoMT in tumour progression and therapy.

## KEY MESSAGES

This review comprehensively summarizes and discusses the phenotypic plasticity of TECs, the critical influence of EndoMT in tumour progression and therapy and potential EndoMT-targeted therapeutic strategies to highlight and connect the gaps in knowledge and therapeutic opportunities.

## 1. Introduction

Tumours are regarded as complicated disorganized organs. They are composed of heterogeneous tumour cells and diverse nontumour stromal components. The latter mainly includes noncellular elements and stromal cells, including vascular cells (e.g. endothelial cells, smooth muscle cells, pericytes), fibrogenic cells (also known as cancer-associated fibroblasts, CAFs), inflammatory cells and immune cells; together, these stromal components constitute the tumour microenvironment (TME), which is suitable for tumour initiation and progression [1–3]. The complicated interactions between tumour cells and stromal cells in the TME greatly affect tumour development, and the latter have gradually become a hotspot in tumour-targeted therapy research [4–6].

Endothelial cells in the tumour microenvironment are also known as tumour endothelial cells or tumour-associated endothelial cells (TECs) [7]. TECs are believed to be a critical stromal cell type in the TME, as they play central roles in tumour angiogenesis and the blood supply, which are necessary for nearly all solid tumours. Thus, antiangiogenic therapy targeting angiogenic factors and their receptors has become an important strategy for tumour treatment [8–10]. Nevertheless, it has gradually been noticed and identified that TECs have phenotypic plasticity. In addition to presenting an endothelial phenotype and participating in tumour neovascularization, TECs also have the potential to transdifferentiate into cells with a mesenchymal phenotype through a process termed endothelial-to-mesenchymal transition (EndoMT).

**Figure 1.**
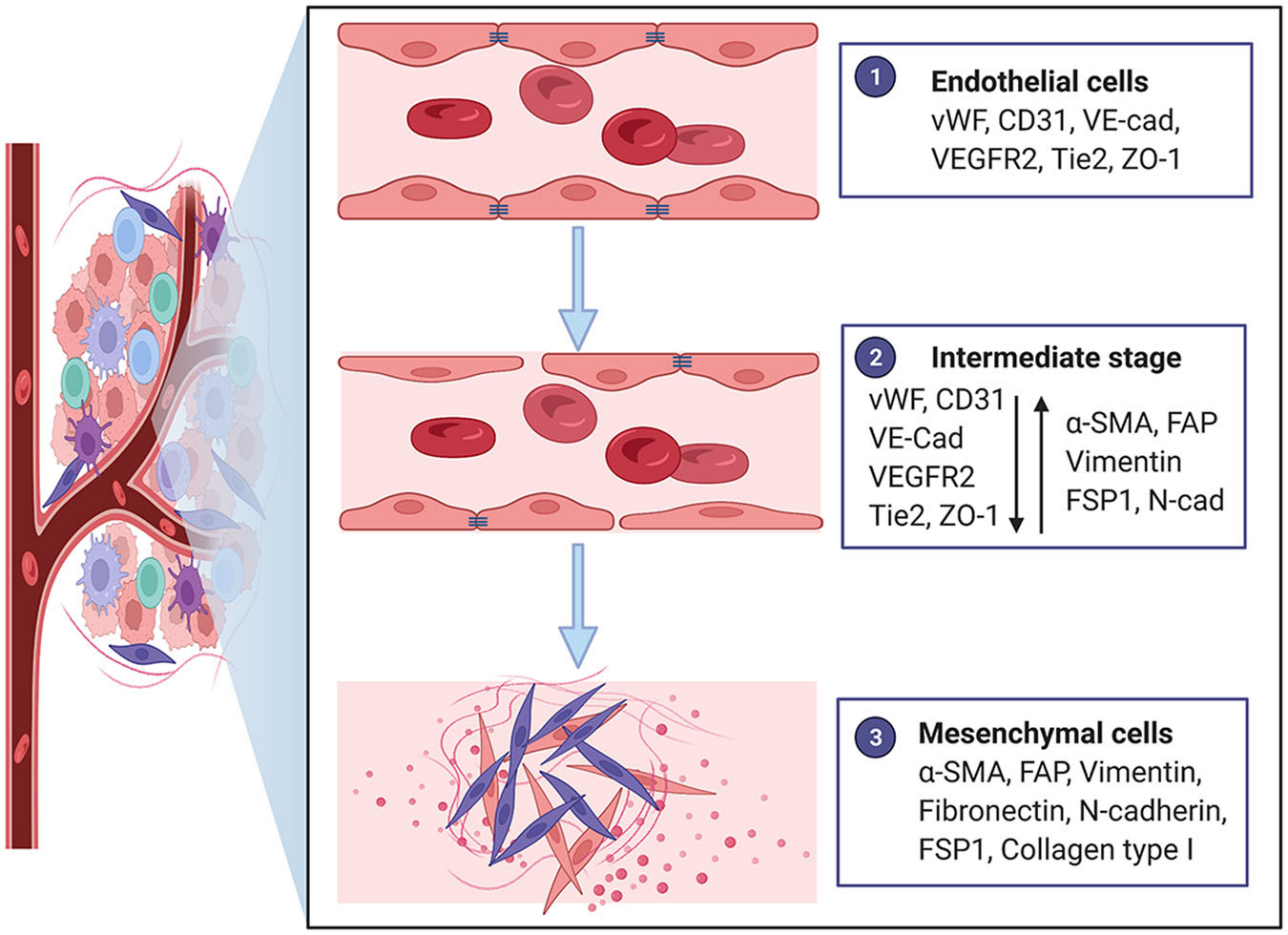
Characteristics of endothelial-to-mesenchymal transition. Morphology changes from an oval shape to a long spindle shape. Gene expression alterations include the loss of endothelial protein marker expression such as von Willebrand factor (vWF), platelet-endothelial cell adhesion molecule-1 (PECAM-1; also known as CD31), vascular-endothelial cadherin (VE-cad), vascular endothelial growth factor receptor 2 (VEGFR2), Tie2 and ZO-1 and the acquisition of mesenchymal marker expression such as α-smooth muscle actin (α-SMA), fibroblast activation protein (FAP), vimentin, fibronectin, N-cadherin (N-cad), fibroblast specific protein 1 (FSP1) and collagen type I. Functional conversion is related to the loss of intercellular junctions and angiogenic capacity, an increase in vascular permeability and the acquisition of migratory and invasive properties. Adapted from ‘Tumor Microenvironment with Callout (Layout)’, by BioRender.com (2022). Retrieved from https://app.biorender.com/biorender-templates.

EndoMT is defined as a cellular transdifferentiation process in which ECs gradually lose endothelial characteristics and acquire a mesenchymal phenotype. It is typically characterized by morphological conversion, gene expression alteration and functional changes in ECs. Morphological conversion refers to the conversion from an oval shape to a spindle-shaped elongated cell morphology. The alteration of gene expression includes reduced expression of endothelium-specific protein markers, such as von Willebrand factor (vWF), platelet-endothelial cell adhesion molecule-1 (PECAM-1; also known as CD31), vascular-endothelial cadherin (VE-cadherin), vascular endothelial growth factor receptor 2 (VEGFR2), Tie2 and ZO-1 and increased expression of mesenchymal protein markers, such as α-smooth muscle actin (α-SMA), fibroblast activation protein (FAP), vimentin, fibronectin, N-cadherin, fibroblast specific protein 1 (FSP1) and collagen type I. Functional changes primarily encompass a loss of intercellular junctions, an increase in vascular permeability, loss of the ability to form blood vessels and acquisition of migratory, invasive and contractile properties (Figure 1) [11]. EndoMT was first reported in physiological heart formation and development, and it has gradually attracted more attention in pathological processes and diseases in recent years, such as organ fibrosis and tumours [12–14]. Although EndoMT has been reported in an increasing number of studies, there is still no unified terminology of EndoMT. The common terms and abbreviations are listed below (Table 1). The unification and standardization of scientific terms is very important for the dissemination and exchange of scientific knowledge, the popularization of scientific achievements and the storage and retrieval of literature. Considering that EndoMT refers to a cellular transdifferentiation process, we named and suggested endothelial-to-mesenchymal transition as terminology to highlight the transition process and suggested EndoMT as an abbreviation based on the prefix.

**Table 1.** The common terms and abbreviations of EndoMT.

Term	Abbreviation	References
Endothelial-to-mesenchymal transition	EndoMT	[12]
Endothelial to mesenchymal transition		[15]
Endothelial-mesenchymal transition		[16]
Endothelial mesenchymal transition	EndMT	[17]
Endothelial-to-mesenchymal transformation		[18]
Endothelial to mesenchymal transformation		[19]
Endothelial mesenchymal transformation	EnMT	[20]
Endothelial-mesenchymal transformation	EMT	[21]
Endothelial-to-myofibroblast transition	End-MyoT	[22]

The mesenchymal transition of TECs will potentially influence their angiogenic ability and thus the effect of antiangiogenic therapy [23], and many studies have found that EndoMT is a pivotal event in tumour progression and is closely associated with tumour growth, angiogenesis, metastasis, immune escape and resistance to treatment [13]; consequently, EndoMT has become an emerging potential therapeutic target for tumour treatment [24]. This review comprehensively summarizes and discusses the phenotypic plasticity of TECs, the critical influence of EndoMT in tumour progression and therapy and potential EndoMT-targeted therapeutic strategies to highlight and connect the gaps in knowledge and therapeutic opportunities.

## 2. EndoMT phenomenon in tumours

EndoMT in tumours was first reported in melanoma-bearing mouse models by Zeisberg et al. in 2007. The mesenchymal markers α-SMA and FSP1 were detected in TECs by immunofluorescence staining of mouse melanoma tissues [25]. Since the first discovery, EndoMT has been gradually identified in a variety of human solid tumour types, including Kaposi’s sarcoma [26], pancreatic cancer [27,28], hepatocellular carcinoma [29], oesophageal adenocarcinoma [30], breast cancer [31], glioblastoma [32], lung cancer [33] and colon cancer [34], by analysing the coexpression of endothelial markers and mesenchymal markers in human tumour tissues (Table 2). What is even more remarkable is the relatively high proportion of TECs undergoing EndoMT (EndoMT ratio, Table 2), which directly reflects the universality of EndoMT in the TME. For instance, Huang et al. indicated that more than 40% of CD31-positive ECs isolated from human glioblastoma tumour tissue specimens expressed FSP1 by flow cytometry analysis [32]. Choi et al. reported that CD31 and α-SMA double-positive cells accounted for nearly 20% of CD31-positive cells in lung cancer tissues according to quantitative analysis of immunofluorescence histochemical double staining [33]. Conversely, in contrast to the universality of EndoMT in various tumour tissues, EndoMT is nonexistent or rare in multiple nontumour tissues (Table 2). For example, unlike in Kaposi’s sarcoma and pancreatic cancer, EndoMT does not occur in normal skin tissues and nontumoural pancreatic tissues [26,28]; Zhu et al. indicated that the rate of EndoMT is extremely low in corresponding normal liver tissues compared with HCC tissues [29]. Intriguingly, Zhang et al. identified CD31 and vimentin double-positive circulating tumour endothelial cells in blood samples from patients with non-small cell lung cancer, which revealed novel evidence and a new form of EndoMT identification [35]. Regardless of the universality of EndoMT in multiple human tumour types or its rarity in corresponding nontumour tissues, these studies all suggest that EndoMT may be a crucial event in tumour progression.

**Table 2.** EndoMT in human tumour tissue specimens.

Tumour type	Tissue specimens	EndoMT identification	EndoMT ratio	Reference
Kaposi’s sarcoma	Kaposi’s sarcoma tissues	Immunofluorescence: broad colocalization of α-SMA and CD31	–	Gasperini et al. [26]
	Normal skin tissues	Immunofluorescence: ɑ-SMA positive cells surrounding CD31-positive endothelium	–	
Pancreatic cancer	Pancreatic tumour tissues	Immunofluorescence: colocalization of CD31 and α-SMA/FSP1/N-cadherin/FAP/SM22ɑ	–	Garcia et al. [28]; Fan et al. [27]
	Nontumoural pancreatic tissues	Immunofluorescence: undetected colocalization of CD31 and α-SMA	–	
Hepatocellular carcinoma(HCC)	HCC tumour tissues	RT–PCR: higher level of ɑ-SMA and FSP1 and lower level of VE-cadherin in CD31^+^ ECs in HCC tumour tissues compared with normal liver tissues	–	Zhu et al. [29]
	Corresponding normal liver tissues		–	
Oesophageal adenocarcinoma(EAC)	EAC tumour tissues	Immunofluorescence: colocalization of FSP1 and CD31	–	Nie et al. [30]
Breast cancer	Breast cancer biopsy tissues	Immunofluorescence: colocalization of CD31 and α-SMA or vimentin	–	Ghiabi et al. [31]
Colon cancer	Colon cancer tissues	Immunofluorescence: colocalization of CD31 and α-SMA	–	Fan et al. [34]
Lung cancer	Lung cancer tissues	Immunofluorescence: colocalization of CD31 and α-SMA	Nearly 20%	Choi et al. [33]
Glioblastoma	Glioblastoma tumour tissues	Immunofluorescence: colocalization of FSP1 and CD31 Western blot: expression of ɑ-SMA and N-cadherin and diminished expression of VEGFR2 in CD31^+^ ECs Flow cytometry: CD31/FSP1 double-positive cells	> 40%	Huang et al. [32]

Identifying EndoMT in human tumour tissues based on the coexpression of relatively specific protein markers of ECs and mesenchymal cells has the following disadvantages. On the one hand, it is only allowed to identify the potential EndoMT and quantify EndoMT ratio, and more comprehensive genomic, transcriptomic and proteomic profiles of any proposed EndoMT phenomenon cannot been provided. Using high-throughput techniques such as quantitative proteomic profiling and single-cell transcriptome analysis of TECs will be beneficial to clarify the global phenotypic plasticity of TECs and the heterogeneity of EndoMT and to discover the possible induction mechanism [36,37]. On the other hand, whether EndoMT cells originate from endothelial cells cannot be determined because of the phenomenon of mesenchymal-to-endothelial transition [38], and a complete transdifferentiation stage cannot be detected due to the probable total loss of endothelium-specific protein markers in newly emerged mesenchymal-like cells. The use of genetic engineering technology to construct a transgenic mouse model to trace cell lineages will effectively overcome the above problems. Using the conventional Cre-LoxP recombination system, Zeisberg et al. constructed Tie2-Cre; R26Rosa-lox-Stop-lox-LacZ transgenic mice to conduct endothelial cell lineage tracing, in which endothelial cells were irreversibly tagged with an expression of the LacZ transgene. They found that approximately 30% of the FSP1^+^ cells in the tumour stroma were β-galactosidase and FSP1 double positive, and 12% of the a-SMA^+^ cells revealed double positivity for a-SMA and β-galactosidase [25]. In another transgenic mouse model, in which endothelial cells were indelibly marked with ZsGreen expression, the colocalization of a-SMA and ZsGreen in TECs was also identified [39]. Combining a cell lineage tracing technique with high-throughput techniques will provide more precise tracing of endothelial cell lineage and facilitate the *in vivo* study of TECs plasticity in tumour progression. Moreover, expression of certain markers is not a reliable sign of a cell type conversion and functional assays must be provided to confirm the identity of the final cell populations. Elucidating functional changes accompanied by gene expression alterations of TECs undergoing EndoMT may intuitively reflect the cell type conversion and biological influence in tumour progression.

## 3. Factors and molecular mechanisms that influence and regulate EndoMT in tumour progression

**Figure 2.**
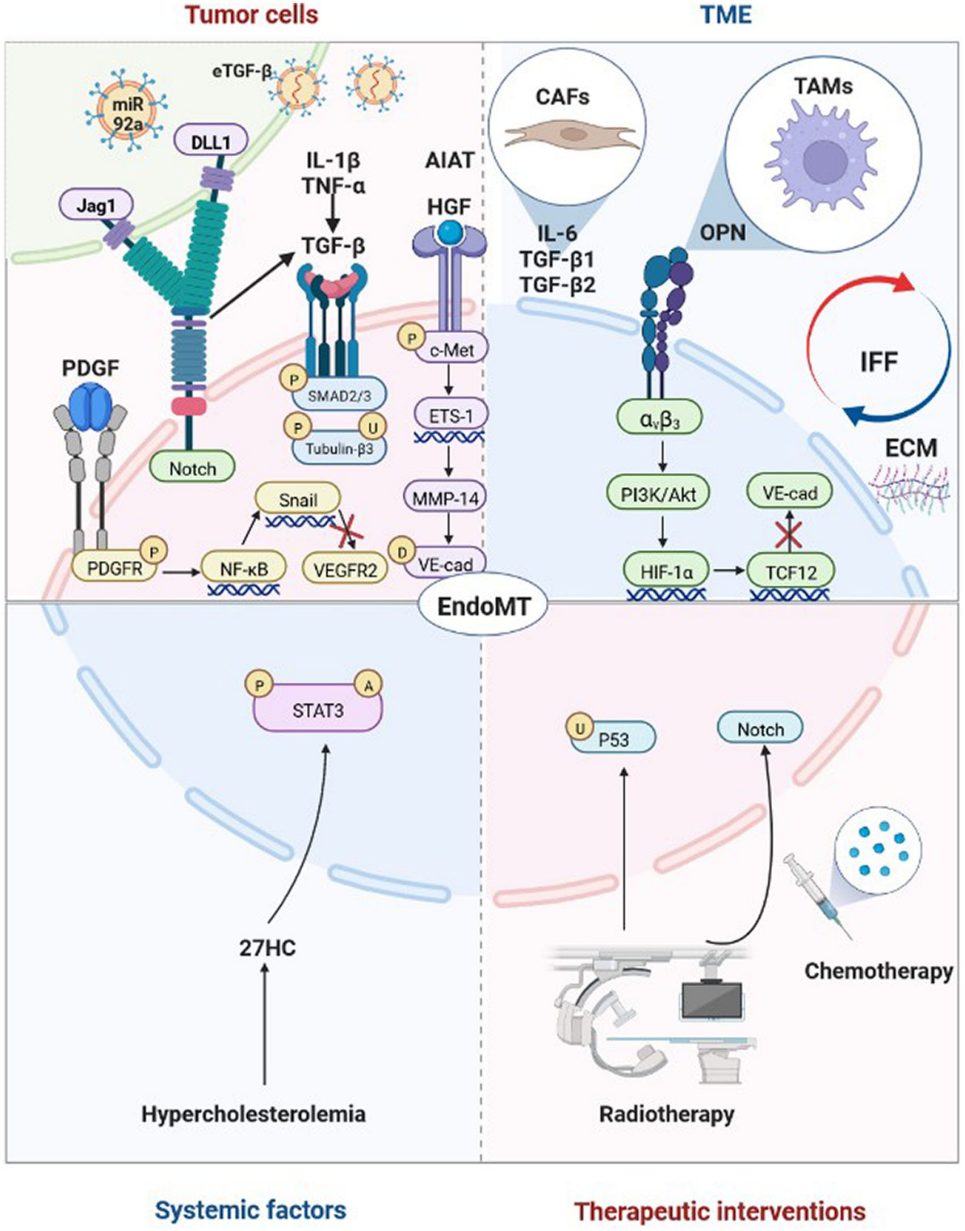
Factors and molecular mechanisms that regulate endothelial-to-mesenchymal transition (EndoMT) in tumour progression. Tumour cells induce EndoMT: Tumour cells activate the Notch pathway *via* Jag1 and DLL1 to induce EndoMT. Tumour cells secrete transforming growth factor-beta (TGF-β), hepatocyte growth factor (HGF), platelet-derived growth factor (PDGF) and α1-antitrypsin (A1AT) to induce EndoMT. TGF-β enhances the phosphorylation of Smad2/3 and the upregulation and phosphorylation of tubulin-β3 to induce EndoMT, and interleukin-1β (IL-1β), tumour necrosis factor-α (TNF-α) and Notch may play synergistic roles. HGF induces the phosphorylation of c-Met, and the expression of ETS-1 enhances the expression of matrix metalloproteinase-14 (MMP-14), which mediates the degradation of vascular-endothelial cadherin (VE-cad). PDGF binds to PDGFR and induces its phosphorylation, which activates NF-κB. Activation of NF-κB upregulates the expression of Snail, thus inhibiting the transcription of vascular endothelial growth factor receptor 2 (VEGFR2). Tumour cells release extracellular vesicles enriched with TGF-β (eTGF-β) and miR-92a to induce EndoMT. The tumour microenvironment (TME) induces EndoMT: Cancer-associated fibroblasts (CAFs) secrete interleukin-6 (IL-6), TGF-β1 and TGF-β2 to induce EndoMT. Tumour-associated macrophages (TAMs) induce EndoMT through the secretion of osteopontin (OPN). OPN-integrin α_v_β_3_ engagement activates the PI3K/Akt pathway and hypoxia-inducible factor-1α (HIF-1α) expression, which in turn transactivates TCF12 gene expression. TCF12 transcriptionally represses the VE-cad gene. Interstitial fluid flow (IFF) and extracellular matrix (ECM) upregulate EndoMT. Systematic factors induce EndoMT: Hypercholesterolemia increases 27-Hydroxycholesterol (27HC) expression and induces EndoMT *via* the acetylation and phosphorylation of STAT3. Therapeutic interventions induce EndoMT: Radiotherapy activates the Notch signalling pathway and upregulates p53 to induce EndoMT. Chemotherapy can also induce EndoMT. Adapted from ‘The Hippo Tumor-suppressor Pathway’, by BioRender.com (2022). Retrieved from https://app.biorender.com/biorender-templates

Mechanistically, although many studies have indicated the influencing factors, molecules and signalling pathways involved in the EndoMT process, mechanistic research involving EndoMT in the context of tumours remains obscure and warrants further investigation. To the best of our knowledge, therapeutic interventions involving radiotherapy and chemotherapy, systemic factors such as hypercholesterolemia, the tumour microenvironment including cancer-associated fibroblasts (CAFs), tumour-associated macrophages (TAMs), interstitial fluid flow (IFF) and extracellular matrix (ECM), and tumour cells may regulate EndoMT in tumour progression (Figure 2).

### 3.1. Tumour cells regulate EndoMT

Considering that tumour cells tend to create a supportive TME by communicating with nontumour stromal components, many studies have focussed on whether they participate in the induction of EndoMT and have elucidated the potential molecular mechanisms. Tumour cells induce EndoMT mainly through direct intercellular contact, secretion of soluble factors and release of extracellular vesicles.

#### 3.1.1. Tumour cells induce EndoMT through direct intercellular contact

Direct contact is one of the classical patterns of intercellular interactions. In this case, cell surface ligands bind to their receptors to activate downstream signalling pathways [40]. EndoMT induced by direct contact between tumour cells and TECs is mainly mediated by activation of the Notch signalling pathway. Breast cancer cells induce EndoMT by direct contact with human umbilical vein endothelial cells (HUVECs), which activates the Jag1/Notch signalling pathway [31]. Glioma cells also activate the Notch signalling pathway and induce EndoMT *via* direct contact with brain ECs, which may be mediated by the DLL1 ligand [24].

#### 3.1.2. Soluble factors secreted by tumour cells induce EndoMT

The secretion of soluble factors by tumour cells that induce EndoMT has been widely reported. The identified effector molecules primarily include transforming growth factor-beta (TGF-β), hepatocyte growth factor (HGF), platelet-derived growth factor (PDGF) and α1-antitrypsin (A1AT).

TGF-β is the most studied effector molecule secreted by tumour cells that induces EndoMT [24,29–31,41–44], and TGF-β1/β2 subtypes have been reported to be closely associated with EndoMT regulation in tumour progression [31,42,43]. Even so, only a few studies have identified the exact molecular mechanisms of TGF-β-induced EndoMT and its activation signalling pathways, such as phosphorylation of Smad2/3 [41] and upregulation and phosphorylation of tubulin-β3 [42]. Interestingly, some studies have indicated that TGF-β alone cannot induce EndoMT effectively and that other cytokines and signalling pathways play synergistic roles in the EndoMT process in tumour progression, such as IL-1β [30], TNF-α [44] and Notch [24,31].

Although TGF-β has been identified in previous studies as the most important effector molecule secreted by tumour cells that induces EndoMT, other potentially secreted factors that may efficiently induce EndoMT have attracted great attention in recent years, such as AIAT [45], HGF/c-Met signalling [32] and the PDGF pathway [23].

#### 3.1.3. Tumour cells release extracellular vesicles to induce EndoMT

Compared with traditional cell interactions, such as direct contact and paracrine interactions, extracellular vesicles, especially exosomes, have gradually received more attention for their role in cell communication [46]. Notably, extracellular vesicles released from tumour cells also have modulatory functions in EndoMT. Yeon et al. reported that exosomes derived from breast cancer cells or mouse melanoma cells induce EndoMT in ECs [47]. Yamada et al. indicated that extracellular vesicles isolated from colon cancer cell-derived conditioned medium induce EndoMT in HUVECs, which may be mediated by miR-92a-3p [48]. In addition, extracellular vesicles may regulate the EndoMT process in the premetastatic niche [49]. Further elucidation of the effector molecules in extracellular vesicles that induce EndoMT would enrich our understanding of the biological function of extracellular vesicles in tumour progression.

It is especially noteworthy that tumour cells do not always induce EndoMT. Omori et al. identified that melanoma cells inhibited EndoMT of TECs by secreting the inflammatory cytokines TNF-α and IL-1 and consequently upregulating lopocalin-type prostaglandin D synthase-derived PDG_2_ [50]. This dual effect of tumour cells on EndoMT regulation deserves further research.

### 3.2. Tumour microenvironment induces EndoMT

TECs are one of the critical stromal cell types in the TME. In addition to communicating with tumour cells, they are influenced concurrently by other stromal components and physical and chemical factors in the TME. CAFs and TAMs were reported to induce EndoMT of TECs in tumour progression. IL-6, TGF-β1 and TGF-β2 secreted by CAFs may be responsible for EndoMT induction [51]. TAMs may induce EndoMT through secretion of osteopontin (OPN). OPN-integrin α_v_β_3_ engagement activates the PI3K/Akt pathway and HIF-1α expression, which in turn transactivates TCF12 gene expression. TCF12 further interacts with EZH2 and histone deacetylases to transcriptionally repress the VE-cadherin gene and thus facilitates EndoMT [34]. Furthermore, interstitial fluid flow (IFF)-induced shear stress and ECM composition may also upregulate EndoMT in tumour progression [19]. Studies investigating EndoMT in the context of the biophysical properties of the TME could be a greater focus in future research.

### 3.3. Systemic factors upregulate EndoMT

Tumours are a systemic disease, and systemic factors may also play critical roles in EndoMT regulation. 27-Hydroxycholesterol (27HC) is an abundant metabolite of cholesterol and induces EndoMT *via* acetylation and phosphorylation of STAT3 [52]. The upregulated EndoMT induced by 27HC suggested that manipulating a high-cholesterol diet and hypercholesterolemia may be systematic therapies for cancer.

### 3.4. Therapeutic interventions induce EndoMT

Excluding local factors, including tumour cells and TME, and systemic factors, therapeutic interventions involving radiotherapy and chemotherapy may induce EndoMT, of which activation of the Notch signalling pathway and upregulation of p53 may result in EndoMT induced by radiotherapy [33,51,53]. Choi et al. reported that knockdown or overexpression of p53 in HUVECs inhibited or increased irradiation-induced expression of EndoMT markers, respectively, such as transcription factors of Snail1, Snail2, Zeb2 implicated in EndoMT and mesenchymal markers of vimentin and α-SMA. Further investigation found that irradiation-induced increased colocalization of α-SMA and CD31 in lung adenocarcinoma-bearing mice was inhibited in EC-specific p53-knockout mice [33]. Banerjee et al. identified that irradiated HUVECs showed reduced expression of VE-cadherin accompanied by increased levels of cleaved Notch1 expression. Moreover, Notch inhibition reduced the colocalization of α-SMA and endomucin increased by irradiation in neuroblastoma-bearing mice [53]. Wawro et al. found that CAF-like cells treated with vincristine combined with conditioned medium obtained from colon cancer cells increased expression of α-SMA, vimentin and contraction proteins and decreased capillary formation ability in human microvascular endothelial cells [51]. Clarifying the molecular mechanisms of EndoMT induced by therapeutic interventions may be beneficial to improving treatment efforts.

In general, although potential influencing factors that regulate EndoMT in tumours have been widely identified, additional efforts to clarify molecules and signalling pathways are still needed.

## 4. EndoMT plays critical roles in tumour progression

**Figure 3.**
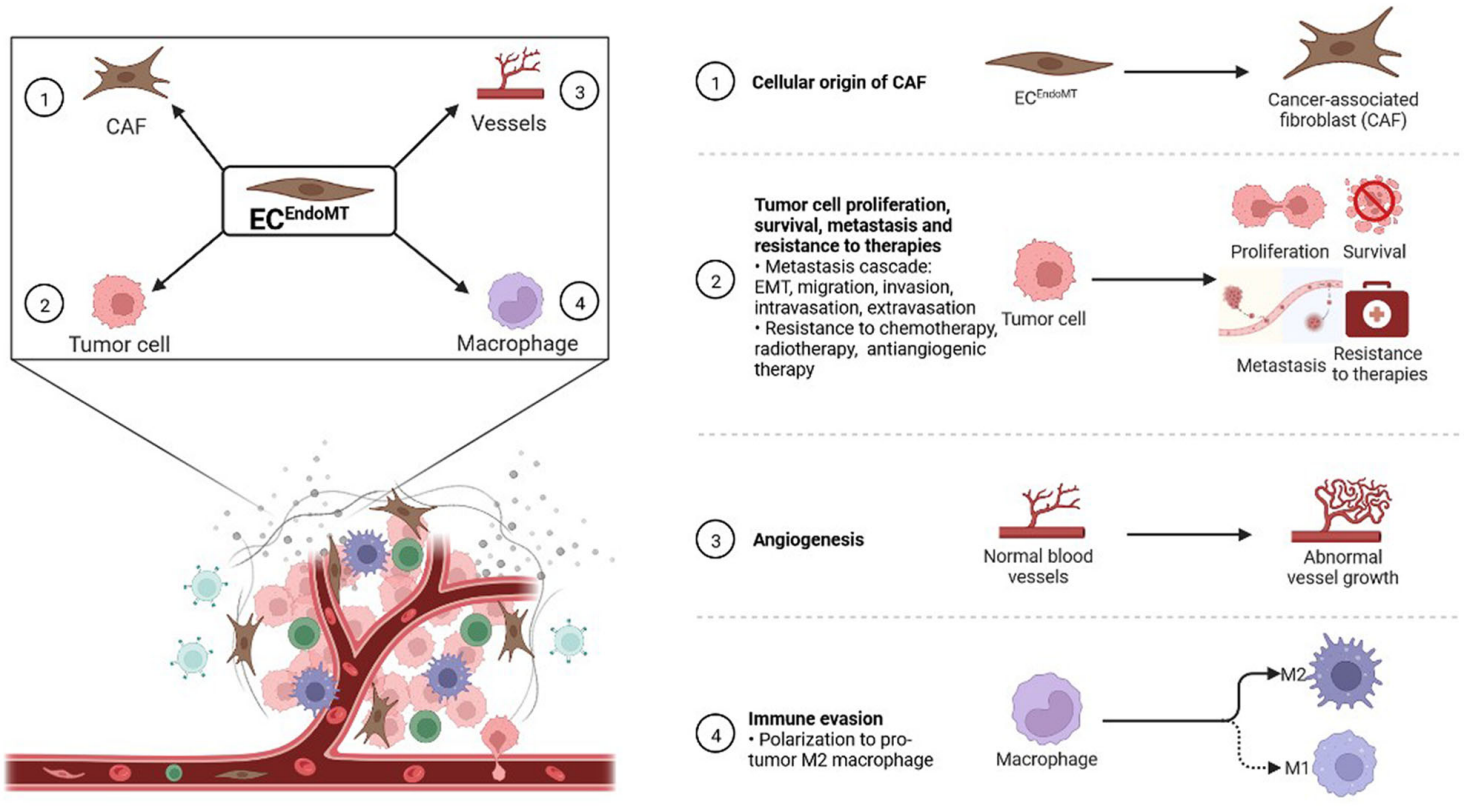
Endothelial-to-mesenchymal transition (EndoMT) promotes tumour progression. **①** Endothelial cell undergoing EndoMT (EC^EndoMT^) mediates the cellular origin of cancer-associated fibroblast (CAF). **②** EC^EndoMT^ promotes tumour cell proliferation, survival and metastasis involving epithelial-mesenchymal transition (EMT), migration, invasion, intravasation and extravasation and resistance to chemotherapy, radiotherapy and antiangiogenic therapy. **③** EC^EndoMT^ induces angiogenesis. **④** EC^EndoMT^ enhances immune evasion by inducing M2 polarization of macrophage. Adapted from ‘The Tumor Microenvironment: Overview of Cancer-Associated Changes’, by BioRender.com (2022). Retrieved from https://app.biorender.com/biorender-templates

Not only the universality of EndoMT in multiple human tumour types but also the diversity of tumour-associated factors that induce EndoMT suggest that EndoMT may be a pivotal event in tumour progression and play critical roles in this process. To date, ECs undergoing EndoMT (ECs^EndoMT^) have been reported to be an important cellular origin of CAFs. ECs^EndoMT^ has also been observed to accelerate tumour growth by promoting tumour cell proliferation, survival and angiogenesis. Moreover, ECs^EndoMT^ may promote tumour metastasis by affecting many key steps, such as tumour cell epithelial-to-mesenchymal transition, migration, invasion, intravasation and extravasation. In addition, ECs^EndoMT^ can also mediate tumour immune escape and resistance to therapies (Figure 3).

### 4.1. EndoMT mediates the origin of CAFs

CAFs represent one of the crucial stromal cell types in the TME that regulate tumour growth, metastasis, immunosuppression and drug resistance [54–56]. The cellular origins of CAFs are largely unknown and mainly include pericytes, vascular smooth muscle cells, endothelial cells that have undergone EndoMT, cancer cells that have undergone epithelial-mesenchymal transition (EMT), tissue residual fibroblasts such as hepatic stellate cells, and bone marrow-derived cells such as mesenchymal stem cells [57]. Among these origins, EndoMT may mediate the significant contribution. For example, the proportion of CAFs originating from EndoMT separately accounts for more than 50% in glioma [32] and 40% in melanoma [25]. Clarifying the contribution of EndoMT to the differentiated origin of CAFs and the influence of cells with various differentiation phenotypes on tumour progression will strengthen recognition of the heterogeneity of CAFs.

### 4.2. EndoMT promotes tumour cell proliferation

One of the distinguishing features of malignant tumours is abnormal cell division and uncontrolled cell proliferation [58]. Clarifying the factors that promote tumour cell proliferation and performing targeted intervention can help inhibit tumour growth. ECs^EndoMT^ may promote tumour cell proliferation, thus accelerating tumour growth in hepatocellular carcinoma, colon cancer and breast cancer [29,31,34]. The exact molecular mechanisms responsible for this promotion process should be further elucidated.

### 4.3. EndoMT enhances tumour cell survival

Tumour cells have an enhanced ability to resist hypoxia, starvation and other harsh conditions caused by tumour overgrowth and therapeutic interventions [58]. Notably, EndoMT may play a critical role in this process. Ghiabi et al. found that breast cancer cells cocultured with ECs^EndoMT^ exhibit enhanced tolerance to starvation and improved survival [31]. This suggests that EndoMT inhibition reduces tumour cell survival and may provide a new approach to tumour treatment.

### 4.4. EndoMT influences tumour angiogenesis

Sustained angiogenesis is one of the classic features of malignant tumours [58] and is a hotspot of tumour-targeted therapy research [59]. Notably, although TECs undergoing EndoMT may partially lose the ability to form blood vessels, they sustain or obtain the ability to synthesize and secrete VEGF and to promote angiogenesis through paracrine signalling [30].

### 4.5. EndoMT is involved in tumour metastasis

Tumour metastasis is a multistep process that mainly starts with tumour cell invasion and migration from primary tumour sites, followed by intravasation, circulation within the bloodstream and extravasation from the vascular system, and finally, colonization and outgrowth at metastatic sites [60]. EndoMT not only affects tumour metastasis at primary tumour sites but also plays an important role at metastatic sites. It has been reported that ECs^EndoMT^ promotes epithelial-to-mesenchymal transition, migration and invasion of tumour cells at the primary tumour site and increased vascular permeability may be beneficial to tumour cell intravasation. Similarly, EndoMT at the metastatic site is beneficial to tumour cell extravasation and may participate in the formation of the premetastatic niche.

#### 4.5.1. EndoMT promotes tumour metastasis at the primary tumour site

Tumour metastasis is a multistep process, and tumour cell invasion and migration are the initial steps of the tumour metastasis cascade. It has been reported that ECs^EndoMT^ can promote the invasion and migration of breast cancer cells [31,44] and colon cancer cells [34], which may be mediated by cell-to-cell contacts and HSP90ɑ secretion, respectively. Notably, the invasion and migration abilities of tumour cells are mainly acquired through the process of EMT [61]. ECs^EndoMT^ can also induce EMT in oral squamous cell carcinoma cells by secreting TGF-β [44].

Increasing vascular permeability caused by EndoMT at primary tumour sites is another potential mechanism for promoting tumour metastasis, which may be beneficial to tumour cell intravasation during metastasis.

#### 4.5.2. EndoMT participates in the formation of the premetastatic niche

In contrast to the extensive interest in EndoMT at primary tumour sites, few studies have focussed on EndoMT within metastatic sites. To our knowledge, EndoMT has not yet been identified in human metastatic tumour tissues. Smeda et al. identified EndoMT of the pulmonary endothelium accompanied by increased vascular permeability at an early stage of metastasis in an orthotopic murine breast cancer model [62]. Krizbai et al. reported that human metastatic melanoma cells induce EndoMT in brain endothelial cells and disrupt monolayer integrity, which presents as reduced transendothelial electrical resistance, enhanced adhesion of metastatic cells to endothelial layers and enhanced transendothelial migration of melanoma cells [63]. Kim et al. found that extracellular vesicles released by breast cancer cells induce EndoMT of liver sinusoidal endothelial cells [49]. These studies suggest that tumour cells tend to induce EndoMT within metastatic sites and that EndoMT may participate in the formation of the premetastatic niche, which may be beneficial to tumour cell extravasation, thus promoting tumour metastasis.

Identifying EndoMT in human metastatic tumour tissues and elucidating whether EndoMT occurs before or after tumour metastasis may increase our understanding of the role of EndoMT in metastasis.

### 4.6. EndoMT mediates tumour immune escape

The immune system functions in immune surveillance, which can lead to the detection and elimination of abnormal components in the body, such as tumour cells caused by genetic mutations. A lack of immune surveillance leads to tumour initiation and progression. Previous studies have found that many factors can induce tumour immunosuppression [64]. Notably, ECs^EndoMT^ can promote tumour immune escape by inducing M2 polarization of macrophages [27,33]. Tumour infiltrating lymphocytes (TILs) are pivotal stromal cell types in the TME and are closely associated with tumour immunoregulation [65]. The origin of CAFs, abnormal angiogenesis, perfusion and permeability mediated by EndoMT may influence the infiltration and function of TILs directly or indirectly [66,67]. Paying more attention to the crosstalk between EndoMT and TILs in further research may expand scientific insight in the field.

### 4.7. EndoMT enhances resistance to therapies

Chemotherapy, radiotherapy and targeted therapy are effective treatments for advanced tumours. However, EndoMT may cause resistance of tumour cells to all these therapies. For example, Huang et al. indicated that EndoMT inhibition significantly enhances the sensitivity of glioma cells to temozolomide [32]. Choi et al. reported that radiotherapy induces EndoMT of TECs, which in turn reduces the therapeutic effects of radiotherapy [33]. In addition, decreased expression of VEGFR2 in TECs undergoing EndoMT renders endothelial resistance to anti-VEGF treatment in glioblastoma [23]. Novel evidence has revealed the existence of CD31 and vimentin double-positive circulating tumour endothelial cells in blood samples from patients with non-small cell lung cancer correlating with poor response to anti-angiogenic therapy and a significantly shortened median progression-free survival, which might be a powerful biomarker for selecting eligible patients and evaluate the anti-angiogenic therapeutic efficacy in clinical practice [35,68]. Paying attention to EndoMT associated with tumour treatment and implementing targeted intervention may enhance the therapeutic effects.

All of the above evidence suggests that EndoMT may promote tumour growth, metastasis, immunosuppression and resistance to therapies, which indicates that more attention should be given to EndoMT. Clarification of the exact molecular mechanisms involved in these promotive effects will accelerate EndoMT-targeted therapy.

## 5. Potential strategies for targeting EndoMT for tumour therapy

EndoMT promotes various malignant biological behaviours of tumours, and the inhibition or reversal of EndoMT during tumour progression may have potential therapeutic effects. Theoretically, inhibiting molecules and signalling pathways responsible for EndoMT induction and maintenance or effector molecules playing roles in the promotion of tumour biological behaviours may effectively weaken tumour progression. Practically, only a few studies have aimed to solve these challenges and mainly focus on identifying the effectiveness of existing drugs. For instance, Chua et al. found that octyl gallate can suppress tumour growth by blocking the effect of HSP90ɑ secreted by ECs^EndoMT^ cells [69]. Nonsteroidal anti-inflammatory drugs (NSAIDs) may suppress and reverse EndoMT induced by vincristine treatment, which suggests that NSAIDs may be useful in preventing chemotherapy-dependent EndoMT [51]. NEO212, a conjugate of temozolomide and perillyl alcohol, can also inhibit and reverse EndoMT caused by glioma cell induction [24]. Interestingly, exosomes derived from mesenchymal stem cells can reverse EndoMT in ECs exposed to tumour cells [47]. Further clarifying the molecules and signalling pathways responsible for EndoMT induction and maintenance or effector molecules playing roles in the promotion of tumour biological behaviours and developing targeted inhibitors will enrich tumour treatment strategies and improve the prognosis of tumour patients.

## 6. Conclusions and prospective

EndoMT is a pivotal event in tumour progression. It has been identified in many human solid tumour types, including Kaposi’s sarcoma, hepatocellular carcinoma, oesophageal adenocarcinoma, breast cancer, glioblastoma, lung cancer, colon cancer and pancreatic ductal adenocarcinoma. Therapeutic interventions involving radiotherapy and chemotherapy, systemic factors such as hypercholesterolemia, the tumour microenvironment including CAFs, TAMs, IFF, and ECM, and tumour cells may regulate EndoMT in tumour progression. Meanwhile, ECs^EndoMT^ contributes to the cellular origin of CAFs, accelerates tumour growth by promoting tumour cell proliferation, survival and angiogenesis, promotes tumour metastasis by affecting many key steps, such as tumour cell epithelial-to-mesenchymal transition, migration, invasion, intravasation and extravasation, and mediates tumour immune escape and resistance to therapies. However, there are relatively few studies on targeting EndoMT for tumour treatment. Providing more comprehensive information on any of the proposed EndoMT phenomena in further research will be beneficial to clarify the global phenotypic plasticity of TECs and the heterogeneity of EndoMT and to discover the possible induction mechanism. Exploring and clarifying the molecular mechanisms responsible for EndoMT induction and maintenance or effector molecules playing roles in the promotion of tumour biological behaviours and developing targeted inhibitors will enrich tumour treatment strategies and improve the prognosis of tumour patients.

## Data Availability

Data sharing is not applicable to this article as no new data were created or analysed in this study
